# Antimicrobial activity of polyhexamethylene biguanide nanoparticles against mastitis-causing *Staphylococcus aureus*

**DOI:** 10.3168/jdsc.2021-0114

**Published:** 2021-07-01

**Authors:** R.F. Leite, J.L. Gonçalves, A. Buanz, C. Febraro, D. Craig, S. Van Winden, L. Good, M.V. Santos

**Affiliations:** 1 Department of Nutrition and Animal Production, School of Veterinary Medicine and Animal Sciences, University of São Paulo, Pirassununga, SP, Brazil, 13635-900; 2 Department of Pharmaceutics, UCL School of Pharmacy, University College London, London, WC1N 1AX, United Kingdom; 3 Department of Pathobiology and Population Sciences, Royal Veterinary College, University of London, London, NW1 0TU, United Kingdom

## Abstract

Postmilking teat disinfection is one of the main measures used to prevent mastitis caused by contagious pathogens, such as *Staphylococcus aureus.* The present study evaluated the antimicrobial activity of polyhexamethylene biguanide (PHMB) and PHMB nanoparticles (NP) against mastitis-causing *Staph. aureus* using a microdilution assay methodology. A total of 20 mastitis-causing *Staph. aureus* isolates were used to determine the minimum inhibitory concentrations (MIC) of PHMB and PHMB NP compared with 3 disinfectants commonly used for teat disinfection (chlorhexidine digluconate, povidone-iodine, and sodium dichloroisocyanurate). The MIC_90_ was defined at the concentrations required to inhibit the growth of 90% of *Staph. aureus*. Our results indicated that PHMB NP presented the lowest MIC value (<0.03 µg/mL) to inhibit 90% of *Staph. aureus*, followed by chlorhexidine digluconate (≥0.25 µg/mL) and PHMB (≥0.5 µg/mL). On the other hand, sodium dichloroisocyanurate (≥500 µg/mL) and povidone-iodine (≥8,000 µg/mL) presented the highest concentrations to inhibit the growth of most *Staph. aureus*. Our preliminary results suggested that both PHMB and PHMB NP have antimicrobial activity against mastitis-causing *Staph. aureus*, which indicates the potential for both to be used as a teat-dip disinfectant to prevent bovine mastitis.

## Highlights

• The PHMB NP were developed by layer-by-layer assembly.
• Mastitis-causing *Staph. aureus* were inhibited by PHMB NP at low concentrations relative to commonly used teat-dip ingredients.
• The NP formulation potentiated the antimicrobial activity of PHMB.

## Graphical Abstract

**Figure:**
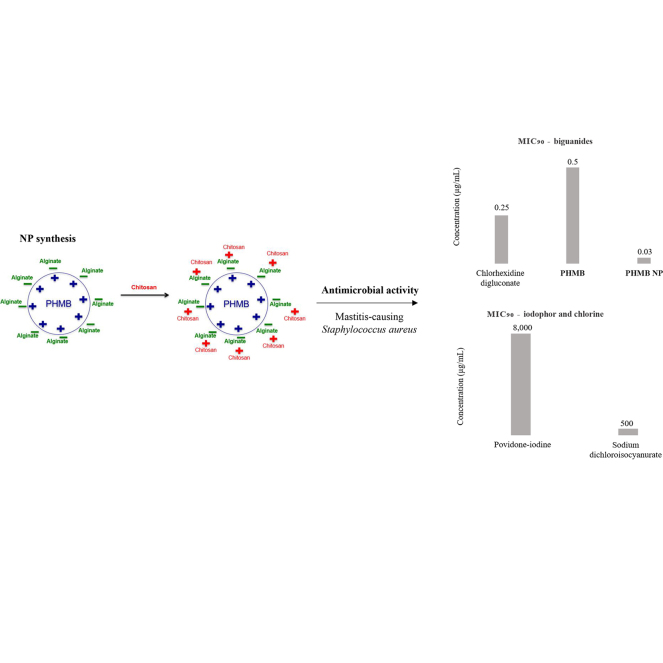
**Summary:** Polyhexamethylene biguanide (PHMB) nanoparticles (NP) developed for this study presented antimicrobial activity against mastitis-causing *Staphylococcus aureus* at lower concentrations than PHMB alone, chlorhexidine digluconate, povidone-iodine, and sodium dichloroisocyanurate. Thus, PHMB NP present potential for the development of new dipping solutions.

*Staphylococcus aureus* is one of the most common pathogens associated with clinical and subclinical mastitis (Hoekstra et al., 2020). Postmilking teat disinfection is a simple, economical, and effective strategy to avoid IMI caused by *Staph. aureus* (Oliver et al., 1990; Berg et al., 2014). However, some drawbacks related to the use of disinfectants on teat skin for mastitis prevention also need to be considered, such as the possibility of milk iodine concentrations higher than 500 µg/L and the risk of teat skin irritation (Burmeister et al., 1998; O'Brien et al., 2013; French et al., 2016). Further, the wide use of a limited range of disinfectants and the increased use of commercial formulations containing low concentrations of the active ingredients may result in bacterial resistance (Maillard, 2013). Monitoring bacterial resistance to disinfectants is essential for monitoring the emergence of resistant pathogens (Davies and Wales, 2019); however, few studies have reported the susceptibility of mastitis-causing pathogens to the disinfectants used for teat disinfection. Polyhexamethylene biguanide (**PHMB**), a cationic polymer that presents a broad antimicrobial spectrum, can bind to DNA and condenses bacterial chromosomes; this mechanism of action may not lead to acquired resistance (Chindera et al., 2016; Sowlati-Hashjin et al., 2020). On the other hand, Müller et al. (2013) reported that PHMB presents a weak interaction with phospholipids from the mammalian cell membrane, and Chindera et al. (2016) reported that once PHMB enters mammalian cells, it is trapped within endosomes and excluded from nuclei. Moreover, previous studies reported the bactericidal and antibiofilm effects of PHMB against virulent *Staph. aureus* (Kamaruzzaman et al., 2016, 2017). Polymer-based nanoparticles (**NP**) are colloidal particles smaller than 1,000 nm composed by a polymeric nucleus that can be loaded with active compounds (Zielińska et al., 2020). Considering that nanotechnology can increase antimicrobial activity and reduce toxicities (Shimanovich and Gedanken, 2016; Wang et al., 2017), our hypothesis was that PHMB NP would inhibit mastitis-causing *Staph. aureus* at lower concentrations than PHMB. Therefore, this study evaluated the antimicrobial activity of PHMB NP, PHMB, and other disinfectants commonly used for teat disinfection [chlorhexidine digluconate (**CHG**), povidone-iodine (**PVP-I**), and sodium dichloroisocyanurate (**NaDCC**)] against *Staph. aureus* isolated from mastitis using the MIC.

The PHMB NP were developed using layer-by-layer assembly (Firdessa et al., 2015; Martínez-Orellana et al., 2020) in association with chitosan and alginate and were evaluated using dynamic light scattering. For NP synthesis, 5 mL of ultrapure water and 2.5 mL of 1 mg/mL PHMB solution in water (Tecrea) were first complexed with 3.75 mL of sodium alginate (grade viscosity 15–25 cP, 1% in H_2_O, Sigma-Aldrich) in water to make a 1:1.5 weight ratio of PHMB to alginate within 50-mL clear-polypropylene conical centrifuge tubes with self-standing bottoms. A polytetrafluoroethylene stirring bar (18 mm) was added to the tubes that were mixed by a magnetic stirrer (Cole-Parmer) at 650 rpm for 20 min and left to stand for another 20 min. A 3.75-mL volume of low-molecular-weight chitosan (50,000–190,000 Da; Sigma-Aldrich) in 1% acetic acid was added to make a 1:1.5 weight ratio of PHMB to chitosan. The ratio of PHMB alginate to chitosan was obtained to be 2.5:1.5 weight ratio in a total volume of 15 mL of solution. The tubes were mixed again by the magnetic stirrer at 650 rpm for 20 min and achieved a final concentration of 167 µg/mL PHMB. Then, NP populations were evaluated by dynamic light scattering to measure particles size, zeta potential polydispersity index, and count rate on a Zetaziser Nano S-90 (Malvern Instruments Ltd.) using DTS1070 cuvettes. The NP presented size <250 nm, polydispersity index <0.5, zeta potential of 60 mV, and count rate of 350 kilocounts/s. Measurements were taken immediately after synthesis and again after 1 wk and 1 mo of storage. No significant changes that could compromise PHMB NP stability, represented by no significant change to their particle size or zeta potential, were observed during storage.

*Staphylococcus aureus* (n = 20) was isolated from mammary quarter milk samples of cows affected by clinical (n = 11; 11 herds) and subclinical (n = 9; 9 herds) mastitis in commercial dairy herds (n = 15) located in Southeast Brazil. These isolates were obtained from previous studies (Leite et al., 2018; Tomazi et al., 2018) and selected due to their resistance (or multiresistance) to antimicrobials commonly used for mastitis treatment (our unpublished data). The *Staph. aureus* isolates were cryopreserved at −80°C in brain heart infusion broth with 20% glycerol. For this study, isolates were retrieved from storage and plated onto blood agar to ensure purity and to confirm their species by MALDI-TOF MS using the direct transfer method (Cameron et al., 2017). Bacterial colonies were analyzed by Microflex LT mass spectrometer (Bruker Daltonics) coupled with Flex Control 3.4 software and MBT Compass 4.1.7 software (Bruker Daltonics). All isolates presented a score of ≥2 for identification as *Staph. aureus*.

The MIC determination of PHMB NP, PHMB, CHG (Rioquimica), PVP-I (Sigma-Aldrich), and NaDCC (Merck and Co.) using the microdilution method described by EUCAST (2019) was adapted and performed in sterile 96-well plates (Cral). Stock solutions of each disinfectant were freshly prepared in sterile ultrapure water at the following concentrations: PHMB and CHG, 20 mg/mL; PVP-I, 64 mg/mL; and NaDCC, 1.4 mg/mL. The PHMB NP were used at the final concentration of 167 µg/mL obtained by laboratory synthesis. For the first column of each plate, we added cation-adjusted Mueller Hinton broth (Becton, Dickinson and Co.) and disinfectants to attain a final volume of 200 µL; in the other columns (2–12), we added 100 µL of Mueller Hinton broth. Serial dilutions were carried out by homogenizing the wells' contents 5 times and transferring 100 µL from the first column to the second using a manual multichannel pipette. After homogenization of the second column, 100 µL was transferred to the third column, and so on until column 12. Therefore, disinfectants were evaluated at the following range of concentrations: free PHMB and CHG, 0.03 to 64 µg/mL; PVP-I, 7.8 to 16,000 µg/mL; NaDCC, 0.24 to 500 µg/mL; and PHMB NP, 0.03 to 66.8 µg/mL. Two microplates were prepared for each isolate, and wells for negative control of broth sterility and positive control of isolate growth were included for each microplate.

Antimicrobial activity of CHG, PHMB, PVP-I, and NaDCC was evaluated against 20 *Staph. aureus* isolates; for PHMB NP, 10 *Staph. aureus* isolates were selected from 10 dairy herds and selected because they displayed multiresistance or resistance to a high number of antimicrobials used for mastitis treatments by in vitro assays, as mentioned above. The *Staph. aureus* isolates were cultured in brain heart infusion broth (Kasvi) and incubated at 37°C for 24 h. Then, bacterial suspensions were standardized at 0.5 McFarland (1 × 10^8^ cfu/mL) using a nephelometer (Uniscience) in 0.9% sterile saline solution and diluted until a final bacterial count of 5 × 10^6^ cfu/mL; they were then immediately used. A volume of 10 µL of standardized bacterial solution was applied in each well (except for broth sterility controls); the plates were covered and homogenized at 200 rpm for 10 min on a stirring table (Quimis) and then incubated at 37°C. After 18 h, 30 µL of 0.05% thiazolyl blue tetrazolium bromide (MTT; Sigma-Aldrich) was added to the plates for MIC determination by visual inspection (Leite et al., 2018).

All analyses were performed in duplicate to score the concentrations needed to result in 50% (**MIC_50_**) and 90% (**MIC_90_**) inhibition. Because disinfectants evaluated in this study belong to different groups of antiseptics in accordance with their chemistry and mode of action, they inhibit pathogen growth at very different concentration ranges (Maillard, 2013). For this reason, disinfectants from the same group were compared considering treatments by 3 major groups: biguanides (PHMB NP, PHMB, and CHG), iodophor (PVP-I), and chlorine (NaDCC). In addition, disinfectants from different groups were compared (all against all). Then, MIC values were evaluated by ANOVA and differences of least squares means using PROC MIXED (SAS version 9.4; SAS Institute Inc.). Differences were considered significant for *P-*values <0.05.

Disinfectant MIC values were significantly different (*P* < 0.0001; Table 1). The PHMB NP inhibited *Staph. aureus* growth at the lowest concentration; on the other hand, the highest MIC values were observed for PVP-I, followed by NaDCC. No differences were observed between MIC_50_ and MIC_90_ for PHMB NP (<0.03 µg/mL), NaDCC (≥500 µg/mL), and PVP-I (≥8,000 µg/mL). However, for CHG and PHMB, MIC_90_ values (≥0.25 and ≥0.5 µg/mL, respectively) were one serial concentration higher than MIC_50_ values (≥0.12 and ≥0.25 µg/mL, respectively). Considering the analysis by disinfectants from the same group, iodophor inhibited 90% of *Staph. aureus* in higher concentrations (≥8,000 µg/mL) than chlorine (*P* = 0.0138) and biguanides (*P* < 0.0001), and inhibitory concentrations obtained for biguanides were significantly lower than those obtained for chlorine (*P* < 0.0001). On the evaluation of biguanides, PHMB NP presented the lowest MIC_90_ (<0.003 µg/mL) to inhibit *Staph. aureus* isolates compared with PHMB (*P* < 0.0001) and CHG (*P* = 0.0036); one serial concentration (*P* < 0.0001) of PHMB (≥0.5 µg/mL) was higher than the concentration obtained for CHG (≥0.25 µg/mL) to determine the MIC_90_. Despite the lower number of *Staph. aureus* isolates tested for PHMB NP, only 1 isolate grew at 0.03 µg/mL; the other 9 isolates did not grow in the concentration ranges that were analyzed. In relation to CHG, it was not possible to determine the MIC for 2 isolates because they did not grow at the lowest concentration evaluated (<0.03 µg/mL), and the other isolates (n = 18) were inhibited by 3 consecutive serial concentrations. For PHMB, 4 concentrations inhibited the growth of *Staph. aureus*.

**Table 1:** Proportion (%) of mastitis-causing *Staphylococcus aureus* isolates inhibited for each tested concentration of disinfectants and MIC_50_ and MIC_90_ values^1^

Disinfectant	MIC (μg/mL)										MIC_50_^2^	MIC_90_^3^
	<0.03	0.06	0.12	0.25	0.5	1	250	500	4,000	≥8,000		
Chlorhexidine digluconate	10	20	50	20	0	0	0	0	0	0	≥0.12	≥0.25
Polyhexamethylene biguanide	0	0	10	50	30	10	0	0	0	0	≥0.25	≥0.5
Polyhexamethylene biguanide nanoparticles^4^	90	10	0	0	0	0	0	0	0	0	<0.03	<0.03
Povidone-iodine	0	0	0	0	0	0	0	0	40	60	≥8,000	≥8,000
Sodium dichloroisocyanurate	0	0	0	0	0	0	40	60	0	0	≥500	≥500

1 In vitro assays considering isolates (n = 20) of *Staph. aureus*.

2 MIC required to inhibit growth of 50% of tested isolates.

3 MIC required to inhibit growth of 90% of tested isolates.

4 A total of 10 *Staph. aureus* isolates were evaluated.

The current study evaluated in vitro antimicrobial activity of PHMB NP against *Staph. aureus* isolates from bovine mastitis. We found that PHMB NP inhibited the growth of 90% of *Staph. aureus* isolates at lower concentrations compared with PHMB, CHG, PVP-I, and NaDCC.

Due to the virulence characteristics of *Staph. aureus*, which often lead to therapy failures, the development of NP technology is considered a potential alternative to overcome the higher microbial resistance of this pathogen (Algharib et al., 2020). In this scenario, some studies have evaluated nano formulations against mastitis-causing *Staph. aureus* for both therapeutic or preventive usage. Orellano et al. (2019) evaluated chitosan NP with a diameter of approximately 138 nm against *Staphylococcus* spp. obtained from mastitis cases. Regarding *Staph. aureus* isolates (n = 3), chitosan NP inhibited their growth at 200 to 400 µg/mL, whereas chitosan concentrations ≥1,600 µg/mL were required to determine the MIC. For CNS (n = 7), inhibitory concentrations of chitosan NP and chitosan varied from 400 to 800 µg/mL and 800 to >1,600 µg/mL, respectively, for most isolates (n = 6). One isolate of *Staph. chromogenes* presented the same MIC value (200 µg/mL) for both compounds. These results described by Orellano et al. (2019) were higher than the MIC values of PHMB NP (<0.03 µg/mL) and free PHMB (≥0.05 µg/mL) against *Staph. aureus* observed in our study.

Chitosan is a biodegradable polymer and is approved as Generally Recognized as Safe by the US Food and Drug Administration (FDA, 2012). Moreover, it has modest antimicrobial activity as well as controlled drug release and mucoadhesive properties (Ali and Ahmed, 2018). For these reasons, it was included in the formulation of the polymer-based NP evaluated in this study. It has been reported that the combination of chitosan and cloxacillin potentiated the antimicrobial activity against 7 CNS isolates from chronic mastitis cases (Breser et al., 2018). Moreover, Ashraf et al. (2012) found that the growth curves of 1 *Escherichia coli* isolate were inhibited by PHMB functionalized silver NP at lower concentrations (0.075–0.15 μg/mL) than PHMB alone (3 μg/mL). Ashraf et al. (2012) suggested that the association of PHMB and silver on an NP formulation potentiated their antimicrobial activity. However, no previous studies evaluated the effects of the association among PHMB, chitosan, and alginate on the potency of antimicrobial activity.

Although the association among chitosan, alginate, and PHMB on the enhancement of antimicrobial activity is unclear, results obtained for PHMB NP highlighted the ability of NP to potentiate antimicrobial activity. Despite belonging to the same antiseptic group, one serial concentration of PHMB (≥0.05 µg/mL) was higher than CHG (≥0.25 µg/mL) to determine MIC_90_. Compared with the results obtained in the present study for CHG (0.25 µg/mL) to inhibit 90% of *Staph. aureus*, Schabauer et al. (2018) found a higher concentration (2 µg/mL) of chlorhexidine diacetate hydrate to determine MIC_90_ for 172 *Staph. aureus* from mastitis cases. After chlorhexidine, the lowest concentration was observed for benzalkonium chloride (4 µg/mL). Considering all disinfectants evaluated by Schabauer et al. (2018), gentisaldehyde (1,000 µg/mL) and 2,3-dihydroxybenzaldehyde (833 µg/mL) presented the highest inhibitory concentrations to determine MIC_90_. These values are lower than those found in our study for NaDCC (≥500 µg/mL). However, Schabauer et al. (2018) reported a lower concentration of iodine (MIC_90_ = 500 µg/mL) to inhibit evaluated isolates compared with the results obtained by us (MIC_90_ = 8,000 µg/mL) and by Azizoglu et al. (2013), who reported an MIC of 1,500 µg/mL to inhibit 29 *Staph. aureus* isolates (78%).

Regarding MIC values, similarities among disinfectants from different classes and differences among disinfectants from the same antiseptic group may be associated with the different methodologies for MIC determination and with the fact that disinfectant formulation varied. In the case of iodine, Azizoglu et al. (2013) evaluated the antimicrobial activity of titratable iodine using the agar dilution technique. On the other hand, microdilution methods were used in our study and in the study by Schabauer et al. (2018). Whereas this study evaluated the antimicrobial activity of PVP-I, Schabauer et al. (2018) evaluated a Lugol's solution containing 5% (wt/wt) iodine and 10% (wt/wt) potassium iodide. Lugol's solution presents free iodine and rapid lethal effects against pathogens, whereas in PVP-I the iodine forms a complex with the polymer povidone, providing a slow and sustained release of iodine that ensures long-lasting efficacy against pathogens (Eggers, 2019). Moreover, PVP-I present a broad spectrum of antimicrobial activity, and it is safe for in vivo usage. Thus, PVP-I is widely evaluated by ex vivo and in vitro studies despite some difficulties related to the interpretation of results due to the paradoxical higher antimicrobial activity with dilutions until a 0.1% strength solution (Lepelletier et al., 2020); this may also explain the high MIC value (≥8,000 µg/mL) obtained in our study.

In conclusion, we observed that PHMB NP presented the lowest concentrations to inhibit the growth of *Staph. aureus* from bovine mastitis cases by in vitro assay. Despite the high antimicrobial activity against *Staph. aureus* isolates, further analysis of the possible toxicity of PHMB NP against mammary epithelial cells and the evaluation of antimicrobial activity using ex vivo and in vivo assays will help further explain the possible benefits of this approach.

## References

[bib1] Algharib S.A., Dawood A., Xie S. (2020). Nanoparticles for treatment of bovine *Staphylococcus aureus* mastitis. Drug Deliv..

[bib2] Ali A., Ahmed S. (2018). A review on chitosan and its nanocomposites in drug delivery. Int. J. Biol. Macromol..

[bib3] Ashraf S., Akhtar N., Ghauri M.A., Rajoka M.I., Khalid Z.M., Hussain I. (2012). Polyhexamethylene biguanide functionalized cationic silver nanoparticles for enhanced antimicrobial activity. Nanoscale Res. Lett..

[bib4] Azizoglu R.O., Lyman R., Anderson K.L. (2013). Bovine *Staphylococcus aureus*: Dose response to iodine and chlorhexidine and effect of iodine challenge on antibiotic susceptibility. J. Dairy Sci..

[bib5] Berg W., Rose-Meierhöfer S., Ammon C., Kobbe C. (2014). Short communication: Dipping efficiency and teat dip residues in milk using an automatic dipping system. J. Dairy Sci..

[bib6] Breser M.L., Felipe V., Bohl L.P., Orellano M.S., Isaac P., Conesa A., Rivero V.E., Correa S.G., Bianco I.D., Porporatto C. (2018). Chitosan and cloxacillin combination improve antibiotic efficacy against different lifestyle of coagulase-negative *Staphylococcus* isolates from chronic bovine mastitis. Sci. Rep..

[bib7] Burmeister J.E., Fox L.K., Hillers J.K., Hancock D.D. (1998). Effects of premilking and postmilking teat disinfectants on teat skin condition. J. Dairy Sci..

[bib8] Cameron M., Barkema H.W., De Buck J., De Vliegher S., Chaffer M., Lewis J., Keefe G.P. (2017). Identification of bovine-associated coagulase-negative staphylococci by matrix-assisted laser desorption/ionization time-of-flight mass spectrometry using a direct transfer protocol. J. Dairy Sci..

[bib9] Chindera K., Mahato M., Kumar Sharma A., Horsley H., Kloc-Muniak K., Kamaruzzaman N.F., Kumar S., McFarlane A., Stach J., Bentin T., Good L. (2016). The antimicrobial polymer PHMB enters cells and selectively condenses bacterial chromosomes. Sci. Rep..

[bib10] Davies R., Wales A. (2019). Antimicrobial resistance on farms: A review including biosecurity and the potential role of disinfectants in resistance selection. Compr. Rev. Food Sci. Food Saf..

[bib11] Eggers M. (2019). Infectious disease management and control with povidone iodine. Infect. Dis. Ther..

[bib12] EUCAST (2019). EUCAST reading guide for broth microdilution. Version 1.0. https://www.eucast.org/ast_of_bacteria/.

[bib13] FDA (2012). GRAS notices. GRN no. 443. Shrimp-derived chitosan. https://www.cfsanappsexternal.fda.gov/scripts/fdcc/index.cfm?set=GRASNotices&id=443&sort=GRN_No&order=DESC&startrow=1&type=basic&search=chitosan.

[bib14] Firdessa R., Good L., Amstalden M.C., Chindera K., Kamaruzzaman N.F., Schultheis M., Röger B., Hecht N., Oelschlaeger T.A., Meinel L., Lühmann T., Moll H. (2015). Pathogen- and host-directed antileishmanial effects mediated by polyhexanide (PHMB). PLoS Negl. Trop. Dis..

[bib15] French E.A., Mukai M., Zurakowski M., Rauch B., Gioia G., Hillebrandt J.R., Henderson M., Schukken Y.H., Hemling T.C. (2016). Iodide residues in milk vary between iodine-based teat disinfectants. J. Food Sci..

[bib16] Hoekstra J., Zomer A.L., Rutten V.P.M.G., Benedictus L., Stegeman A., Spaninks M.P., Bennedsgaard T.W., Biggs A., De Vliegher S., Mateo D.H., Huber-Schlenstedt R., Katholm J., Kovács P., Krömker V., Lequeux G., Moroni P., Pinho L., Smulski S., Supré K., Swinkels J.M., Holmes M.A., Lam T.J.G.M., Koop G. (2020). Genomic analysis of European bovine *Staphylococcus aureus* from clinical versus subclinical mastitis. Sci. Rep..

[bib17] Kamaruzzaman N.F., Chong S.Q.Y., Edmondson-Brown K.M., Ntow-Boahene W., Bardiau M., Good L. (2017). Bactericidal and anti-biofilm effects of polyhexamethylene biguanide in models of intracellular and biofilm of *Staphylococcus aureus* isolated from bovine mastitis. Front. Microbiol..

[bib18] Kamaruzzaman N.F., Firdessa R., Good L. (2016). Bactericidal effects of polyhexamethylene biguanide against intracellular *Staphylococcus aureus* EMRSA-15 and USA 300. J. Antimicrob. Chemother..

[bib19] Leite R.F., Gonçalves J.L., Peti A.P.F., Figueiró F.S., Moraes L.A.B., Santos M.V. (2018). Antimicrobial activity of crude extracts from actinomycetes against mastitis pathogens. J. Dairy Sci..

[bib20] Lepelletier D., Maillard J.Y., Pozzetto B., Simon A. (2020). Povidone iodine: Properties, mechanisms of action, and role in infection control and *Staphylococcus aureus* decolonization. Antimicrob. Agents Chemother..

[bib21] Maillard J.-Y., Fraise A.P., Maillard J.-Y., Sattar S.A. (2013). Russell, Hugo and Ayliffe's Principles and Practice of Disinfection, Preservation and Sterilization.

[bib22] Martínez-Orellana P., Baxarias M., Good L., Solano-Gallego L. (2020). The effects of polyhexamethylene biguanide (PHMB) and TLR agonists alone or as polyplex nanoparticles against leishmania infantum promastigotes and amastigotes. Vet. Sci..

[bib23] Müller G., Koburger T., Kramer A. (2013). Interaction of polyhexamethylene biguanide hydrochloride (PHMB) with phosphatidylcholine containing o/w emulsion and consequences for microbicidal efficacy and cytotoxicity. Chem. Biol. Interact..

[bib24] O'Brien B., Gleeson D., Jordan K. (2013). Iodine concentrations in milk. Ir. J. Agric. Food Res..

[bib25] Oliver S.P., King S.H., Lewis M.J., Torre P.M., Matthews K.R., Dowlen H.H. (1990). Efficacy of chlorhexidine as a postmilking teat disinfectant for the prevention of bovine mastitis during lactation. J. Dairy Sci..

[bib26] Orellano M.S., Isaac P., Breser M.L., Bohl L.P., Conesa A., Falcone R.D., Porporatto C. (2019). Chitosan nanoparticles enhance the antibacterial activity of the native polymer against bovine mastitis pathogens. Carbohydr. Polym..

[bib27] Schabauer A., Zutz C., Lung B., Wagner M., Rychli K. (2018). Gentisaldehyde and its derivative 2,3-dihydroxybenzaldehyde show antimicrobial activities against bovine mastitis *Staphylococcus aureus*. Front. Vet. Sci..

[bib28] Shimanovich U., Gedanken A. (2016). Nanotechnology solutions to restore antibiotic activity. J. Mater. Chem. B Mater. Biol. Med..

[bib29] Sowlati-Hashjin S., Carbone P., Karttunen M. (2020). Insights into the polyhexamethylene biguanide (PHMB) mechanism of action on bacterial membrane and DNA: A molecular dynamics study. J. Phys. Chem. B.

[bib30] Tomazi T., Ferreira G.C., Orsi A.M., Gonçalves J.L., Ospina P.A., Nydam D.V., Moroni P., dos Santos M.V. (2018). Association of herd-level risk factors and incidence rate of clinical mastitis in 20 Brazilian dairy herds. Prev. Vet. Med..

[bib31] Wang L., Hu C., Shao L. (2017). The antimicrobial activity of nanoparticles: Present situation and prospects for the future. Int. J. Nanomedicine.

[bib32] Zielińska A., Carreiró F., Oliveira A.M., Neves A., Pires B., Venkatesh D.N., Durazzo A., Lucarini M., Eder P., Silva A.M., Santini A., Souto E.B. (2020). Polymeric nanoparticles: Production, characterization, toxicology and ecotoxicology. Molecules.

